# Facets of Arctic energy accumulation based on observations and reanalyses 2000–2015

**DOI:** 10.1002/2016GL070557

**Published:** 2016-10-09

**Authors:** Michael Mayer, Leopold Haimberger, Marianne Pietschnig, Andrea Storto

**Affiliations:** ^1^ Department of Meteorology and GeophysicsUniversity of ViennaViennaAustria; ^2^ Austrian Polar Research InstituteViennaAustria; ^3^ Centro Euro‐Mediterraneo sui Cambiamenti ClimaticiBolognaItaly

**Keywords:** Arctic, energy budget, climate change, ocean heat content, sea ice

## Abstract

Various observation‐ and reanalysis‐based estimates of sea ice mass and ocean heat content trends imply that the energy imbalance of the Arctic climate system was similar [1.0 (0.9,1.2) Wm^−2^] to the global ocean average during the 2000–2015 period. Most of this extra heat warmed the ocean, and a comparatively small fraction went into sea ice melt. Poleward energy transports and radiation contributed to this energy increase at varying strengths. On a seasonal scale, stronger radiative energy input during summer associated with the ice‐albedo feedback enhances seasonal oceanic heat uptake and sea ice melt. In return, lower sea ice extent and higher sea surface temperatures lead to enhanced heat release from the ocean during fall. This weakens meridional temperature gradients, consequently reducing atmospheric energy transports into the polar cap. The seasonal cycle of the Arctic energy budget is thus amplified, whereas the Arctic's long‐term energy imbalance is close to the global mean.

## Key Points

- Arctic energy imbalance was close to global mean imbalance during 2000–2015
- Atmospheric and oceanic transports and radiative energy input contributed to imbalance at varying strengths
- Seasonal trends reveal amplification of the annual cycle of the Arctic energy budget over the past 15 years

## 1 Introduction

The Arctic climate system experienced profound changes over the past decades, the most prominent of which are the record low sea ice extent minima in the years 2007, 2011, and 2012. Several modeling studies showed that these changes have profound impact on the Arctic's energy budget. Besides a long‐term positive energy imbalance, these changes act to amplify its annual cycle, including net radiation at top‐of‐the‐atmosphere (Rad_TOA_), ocean heat content (OHC), and energy going into ice melt [*Bintanja and Van der Linden*, 2013; *Carton et al*., 2015; *Semmler et al*., 2012] and to reduce atmospheric energy convergence during fall [*Tietsche et al*., 2011].

A number of observation‐based studies assessing Arctic climate change in an energy budget context exist [e.g., *Kay and L*'*Ecuyer*, 2013; *Rudels et al*., 2015; *Krikken and Hazeleger*, 2015], but these usually focus on one domain rather than the coupled atmosphere‐ocean‐sea ice energy budget. For example, *Hartmann and Ceppi* [2014] used satellite‐derived data to document seasonal changes in Rad_TOA_ during the 2000–2013 period, finding a strong signature of the ice‐albedo feedback leading to increased absorption of solar radiation in summer. They computed implied changes in poleward energy transports but did not consider variations in the ocean energy storage rate.

Here we aim at filling this gap. We use satellite data, state‐of‐the‐art atmospheric reanalyses, as well as coupled and uncoupled ocean and sea‐ice reanalyses to assess recent changes in the coupled atmosphere‐ocean‐sea ice energy budget of the Arctic. After a description of employed data and methods (section 2), we investigate the energy accumulation in the Arctic cap (section 3). Then we explore the temporal variability of the coupled budget (sections 4 and 5) and finally examine seasonal trends of relevant budget terms (section 6). The results are discussed in section 7.

## 2 Methods and Data

The vertically integrated energy budget of the atmosphere reads as

(1) $$F_{S}\;=\;{\text{Rad}}_{TOA}‐\nabla \cdot F_{A}‐AET\text{,}$$

where F_S_ is net surface energy flux (positive downward), − ∇·F_A_ is the convergence of lateral atmospheric energy transports, and AET is the rate of atmospheric energy storage.

The vertically integrated energy budget of the ocean including sea ice reads as

(2) $$F_{S}\;=\;\text{OHCT}\;+\;\text{MET}\;+\;\nabla \cdot F_{O}\;+\;\nabla \;\cdot F_{I}\text{,}$$

where OHCT is the tendency of ocean heat content, MET represents the tendency of sea ice melt enthalpy, and ∇·F_O_ and ∇·F_I_ denote the divergence of vertically integrated transports of ocean and ice enthalpy, respectively.

Consistent with earlier works [e.g., *Overland and Turet*, 1994; *Porter et al*., 2010], the area of study is the oceanic area north of 70°N. Grid points over land are masked out for area‐averages so that equations (1) and (2) are consistent. Combining equations (1) and (2) and introducing the area‐averaging operator for the ocean‐covered area north of 70°N {..} yields the coupled energy budget of the Arctic cap:

(3) $$\left\{{\text{Rad}}_{TOA}\right\}-\left\{\nabla \cdot F_{A}\right\}‐\left\{\nabla \cdot F_{O}\right\}‐\left\{\nabla \cdot F_{I}\right\}=\left\{AET\right\}+\left\{\text{OHCT}\right\}+\left\{\text{MET}\right\}$$

In practice equation (3) is not exactly satisfied when employing different input data sets for the different terms, but the budget residual is reasonably small as will be discussed.

We employ satellite‐measured and flux‐adjusted Rad_TOA_ data from Clouds and Earth's Radiant Energy System (CERES) [*Wielicki et al*., 1996] and compute mass‐adjusted ∇·F_A_ as well as AET from European Centre for Medium‐Range Weather Forecasts Interim Re‐Analysis (ERA‐I) [*Dee et al*., 2011] and the Japan Meteorological Agency 55 year reanalysis (JRA55) [*Kobayashi et al*., 2015] data following the method of *Mayer and Haimberger* [2012]. The agreement between the ∇·F_A_ estimates from ERA‐I and JRA55 is very good with *r* = 0.96 for the polar cap monthly anomalies. Hence, the mean of the two data sets is used throughout this paper as a best estimate for this term.

The net surface energy flux is computed as a residual from the terms on the right in equation (1). Moreover, we employ a composited direct net surface flux estimate using CERES EBAF 2.8 shortwave radiation [*Kato et al*., 2013], ERA‐I longwave radiation, and turbulent fluxes over ice, as well as OAflux [*Jin and Weller*, 2008] turbulent fluxes over ocean. Rationale for this choice is given in Text S1 in the supporting information.

Oceanic enthalpy transports are obtained from coupled ocean‐sea‐ice reanalysis CGLORS025V5 at ¼° resolution (C‐GLORS) [*Storto et al*., 2016]. Computations are performed on the native tripolar grid using daily fields. We use Gauss's theorem to obtain Arctic average−∇·F_O_ from zonally integrated transports. We neglect the effect of river runoff on ∇·*F_O_*.

Ocean heat content is obtained from C‐GLORS and objective ocean temperature analysis UK Met Office Hadley Centre EN4 [*Good et al*., 2013]. The number of observations going into EN4 exhibits a strong annual cycle (see Figure S2) due to the varying sea ice cover, leading to a maximum of available observations in fall. This induces spurious monthly OHC tendencies, and hence, only fall OHC from EN4 will be presented. We additionally employ OHC estimates from the uncoupled Ocean Reanalysis System 4 (ORAS4) [*Balmaseda et al*., 2012].

Ice enthalpy changes through ice temperature variations are small [*Serreze and Barry*, 2014] and neglected and so is the effect of snow. Hence, the tendency of sea ice melt enthalpy is proportional to the sea ice melt rate:

(4) $$\text{MET}\;=\;d/dt\left(\text{ME}\right)=\;-d/dt\;\left(L_{f}*m_{\text{ice}}\right)\text{,}$$

where L_f_ is latent heat of freezing (0.33 × 10^6^ J kg^−1^) and m_ice_ is sea ice mass. Note the sign convention so that positive F_S_ goes with positive MET (see equation (2)), which, in fact, means sea ice loss (see equation (4)). Sea ice mass is computed from C‐GLORS and Pan‐Arctic Ice Ocean Assimilation System (PIOMAS) [*Schweiger et al*., 2011]. Although sea ice volume from PIOMAS is generally viewed as a reference estimate [see, e.g., *Chevallier et al*., 2016], we found a shift in PIOMAS summer ice volume in 2008/2009. This shift is coincident with a discontinuity in sea ice fraction analyses from PIOMAS, which is not present in C‐GLORS. A likely reason for this discrepancy is the fact that PIOMAS employs an operational near‐real‐time sea ice fraction product (A. Schweiger, personal communication, 2016), while C‐GLORS employs a delayed‐time sea ice fraction product, which is designed to provide consistent time series. Please see Text S3 [*Nolin et al*., 1998; *Cavalieri et al*., 1996; *Peng et al*., 2013; *Andersen et al*., 2007; *Lindsay and Zhang*, 2006] for details. Hence, we prefer C‐GLORS when investigating sea ice mass on subannual scales.

Sea ice transport out of the Arctic is based on a small mean value in terms of energy (about 3 Wm^−2^ according to *Serreze et al*. [2007]). This value is even smaller for the region considered here, with a yearly mean {− ∇·F_I_} of about 0.5 Wm^−2^ and a January maximum of 1.7 Wm^−2^ (based on C‐GLORS). Hence, even a halving of sea ice volume transport across 70°N over 2000–2015 would yield a maximum seasonal trend of only about −0.6 Wm^−2^ per decade, which is likely an overestimation given the opposing effects of decreasing ice thickness [*Hansen et al*., 2013] and increased sea ice area export [*Smedsrud et al*., 2011] in recent years. Thus, since the observed decadal changes in F_I_ are probably small and the variability of F_I_ is difficult to reliably quantify, we neglect this term in our diagnostics.

Monthly tendencies of OHC and ME are computed from centered differences of monthly means of the respective fields. Monthly anomalies mean that the annual cycle has been removed. Seasonal trends are computed using ordinary least squares (presented in section 6) or median of pairwise slopes (in supporting information). The period of study is March 2000 to February 2015, given by availability of CERES data.

## 3 Changes in Energy Content 2000–2015

**Figure 1:**
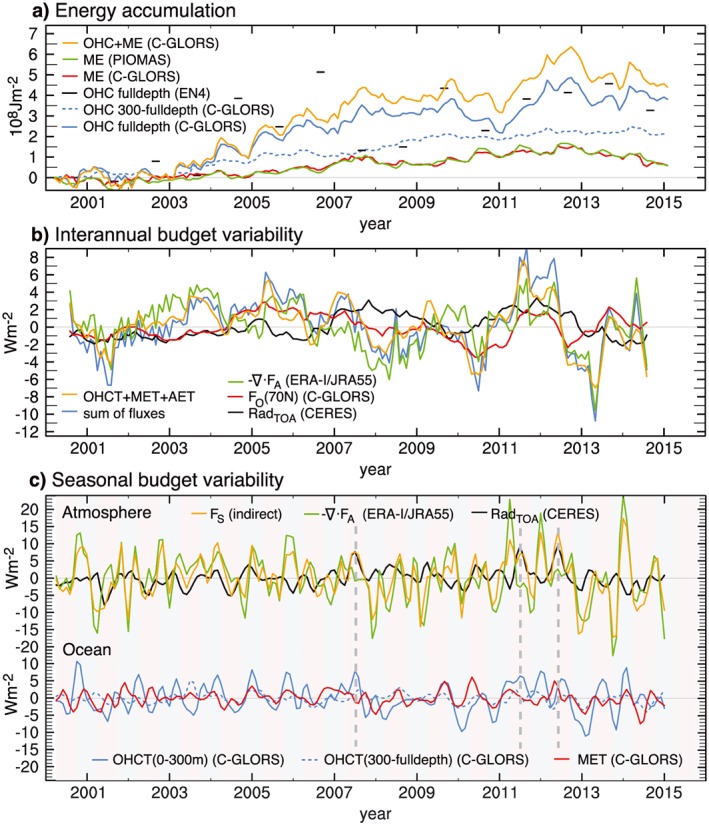
(a) Accumulated energy increase in the Arctic (ocean‐covered area north of 70°N) as estimated from ocean heat content (OHC) over various depths and required melt enthalpy (ME) based on various data sets; (b) comparison of interannual variations of total Arctic energy storage rate (atmosphere, sea ice, and full‐depth ocean) and anomalous net radiation at TOA, atmospheric and oceanic energy convergence, and the sum of these fluxes (12 month running mean); (c) anomalous Rad_TOA_, atmospheric energy convergence, net surface energy flux, OHC tendency, and ME tendency in the Arctic (monthly anomalies with a 1‐2‐1 filter applied). Red and blue shadings indicate warm (April–September) and cold (October–March) seasons, respectively. Vertical dashed lines indicate summers of years with very low minimum sea ice extent.

The time evolution of energy content in the Arctic Ocean based on various data sets is presented in Figure 1a. A notable feature of the full‐depth OHC curve from C‐GLORS is a steady increase up to 2006 and strong OHC increase during the years of exceptionally low autumn sea ice extent (2007, 2011, and 2012). For example, the OHC increase during the 2011–2012 period of about 1 × 10^8^ Jm^−2^ corresponds to a warming rate of more than 3 Wm^−2^. After 2013, ice mass recovered as documented by *Tilling et al*. [2015], but OHC remained at high 2012 levels. Interannual variations of ocean warming are mainly confined to the layers above 300 m, while the OHC increase below is quite steady (see Figure 1a). Over the 15 year period, full‐depth ocean heat content from C‐GLORS increased by about 3.8 × 10^8^ Jm^−2^ corresponding to a heating rate of about 0.8 Wm^−2^. The OHC increase in the upper 300 m of the ocean is only half of this value (0.4 Wm^−2^), suggesting that ocean heat transport at greater depths contributed significantly to the observed total OHC increase, but vertical redistribution possibly also played a role [*Tietsche et al*., 2016]. We compare OHC evolution from C‐GLORS and EN4 for the fall season (September–November mean), when the number of observations assimilated in EN4 is generally highest. The average OHC increase during the 2000–2015 period is very similar in C‐GLORS and EN4, but considerable discrepancies exist during the 2005–2009 period, probably owing to variability in observation sampling affecting EN4. Ocean heat content increase from ORAS4 (not shown) is in very good agreement with C‐GLORS, with an average Arctic OHCT of 1.1 Wm^−2^ for the 2000–2015 period. Thus, the long‐term OHC evolution is consistent in the three employed products (two dynamical reanalyses and one objective analysis), unlike climate model‐based estimates [*Boé et al*., 2009].

Energy going into sea ice melt as estimated from C‐GLORS amounted to 0.5 × 10^8^ Jm^−2^, equivalent to an average energy input of 0.14 Wm^−2^. This value is in excellent agreement with the PIOMAS estimate (0.14 Wm^−2^). Thus, while the seasonal energy input to the Arctic Ocean is approximately equally partitioned between OHCT and MET [*Serreze et al*., 2007] long‐term energy accumulation clearly goes into warming the ocean instead of melting sea ice (0.81 versus 0.14 Wm^−2^). Figure 1a also reveals excessive summer melt in PIOMAS from 2009 onward (note the wiggles compared to C‐GLORS MET), which is likely spurious (see section 2 and Text S3). Hence, we exclude PIOMAS from further diagnostics on the subannual scale.

In sum, OHC increase, sea ice melt, and the small atmospheric energy storage rate (<0.1 Wm^−2^) imply an energy imbalance of the Arctic of about 1.0 (0.9,1.2) Wm^−2^. This value is very similar to the global mean time‐averaged OHCT from C‐GLORS, EN4, and ORAS4 of 1.1 (1.0,1.2) Wm^−2^ over the 2000–2015 period. When considered with respect to the total surface of the Earth (~0.7 Wm^−2^), our storage rate estimate is in very good agreement with the recent estimate of Earth's energy imbalance by *Llovel et al*. [2014] (0.64 ± 0.44 W m^−2^ for the 2005–2013 period). This result implies that the observed Arctic surface warming, faster than the global average (Arctic amplification), is not reflected in an energy imbalance above the global average.

## 4 Drivers of Energy Accumulation

To explore the varying contribution of {‐ ∇·F_A_}, {‐ ∇ ⋅ F_O_}, and {Rad_TOA_} to the mean Arctic energy imbalance, we present monthly anomalies of these terms along with their sum as well as the storage rate with a 12 month running mean applied in Figure 1b. The general agreement between the sum of fluxes and storage rate is very good (*r* = 0.86), but the standard deviation of the fluxes is higher than that of the storage rate (3.4 and 2.7 Wm^−2^, respectively), yielding a residual standard deviation of 1.5 Wm^−2^. This good agreement also indicates that accumulated energy fluxes into the Arctic track ocean energy changes quite well (see Figure S4). Please note that neither CERES radiation nor ∇·F_A_ is used as input to the C‐GLORS reanalysis, i.e., variability of energy fluxes into the Arctic and the Arctic Ocean storage rate are largely independent.

Considering the three flux terms separately, the standard deviation of lateral atmospheric energy transports is larger than that of oceanic transports and net radiation at TOA (2.7, 1.4, and 1.4 Wm^−2^, respectively).

Ocean heat transport seems to have substantially contributed to the OHC increase during 2005–2007 (see Figure 1b). This is in agreement with the results of *Schauer and Beszczynska‐Möller* [2009] finding increased temperature transport through Fram Strait at that time, but heat transport variations through the Barents Sea Opening are equally important [see, e.g., *Årthun et al*., 2012; *Lique and Steele*, 2013].

Anomalies of Rad_TOA_ are generally negative before 2007 and positive afterward. This indicates that this term significantly contributed to ocean warming after 2006, especially during 2011–2012, representing the contribution from the ice‐albedo feedback.

In fact, all three flux terms show positive anomalies during the 2011–2012 period, adding up to +8 Wm^−2^. Atmospheric energy convergence was anomalously low during winter 2012/2013 (down by −10 Wm^−2^), which is consistent with observed sea ice mass recovery at that time.

## 5 Budget Variability on Seasonal Scale

To explore seasonal variability of the Arctic energy budget, we present monthly anomalies of polar cap area averages of F_S_ and its contributors, Rad_TOA_ and − ∇ ⋅ F_A_, with a 1‐2‐1 time filter applied in Figure 1c. Largest {Rad_TOA_} anomalies occur in summer, with positive net radiation anomalies during the summers of 2007, 2011, and 2012, which is a result of increased absorption due to reduced sea ice cover. Consequently, negative summer anomalies of {Rad_TOA_} are found for the early 2000s, when sea ice extents were still relatively large compared to more recent years.

Convergence of atmospheric energy transports shows a less clear pattern of seasonal anomalies when compared to net radiation. Strong positive energy convergence (i.e., energy import) anomalies are found during middle to end of recent winters (2010/2011, 2011/2012, and 2013/2014). In contrast, during fall of 2007, 2011, and 2012, negative convergence anomalies can be seen from Figure 1c. This decrease of atmospheric energy import during early winter will be explored in more detail later. Variability of {−∇ ⋅ F_A_} in summer is generally lower compared to winter, which is related to the climatologically weaker temperature gradients in summer. Overall, {−∇ ⋅ F_A_} exhibits a higher variability compared to Rad_TOA_ (standard deviations are 7.4 Wm^−2^ and 2.7 Wm^−2^, respectively).

Anomalous net radiation and horizontal energy convergence are balanced by net surface energy flux anomalies (and comparatively small AET variations). Inspection of the time series in Figure 1c shows positive surface flux and associated ice melt anomalies during the exceptional summers of low sea ice extent. These {F_S_} anomalies were linked to positive area‐averaged net radiation anomalies (Figure 1c), which in turn were dominated by longwave (shortwave) radiation anomalies in the early (high) summer [see *Kapsch et al*., 2013]. However, atmospheric transports also play a role in modulating {F_S_}, most notably during winter, e.g., in 2013/2014.

Arctic average OHCT (Figure 1c) exhibits considerable anomalies, including an obvious trend: negative (positive) {OHCT} anomalies dominate in summer (winter) during the early 2000s, while the opposite is the case in more recent years. This suggests that the annual cycle of {OHCT} has amplified over the considered period and will be discussed in detail in section 6. Figure 1c also reveals that in agreement with *Tietsche et al*. [2016], the upper 300 m of the ocean dominate {OHCT} (note the low variance of {OHCT} below 300 m as shown by the dashed blue curve), in contrast to the long‐term OHC trends to which the deeper layers contribute roughly 50% (see section 4).

Variability of energy going into ice melt (Figure 1c) is lower than {OHCT} variability with respective standard deviations of 2.1 Wm^−2^ and 4.8 Wm^−2^ (4.2 Wm^−2^ for the upper 300 m {OHCT}; values based on C‐GLORS). Reasons for this are the higher albedo of sea ice making it less susceptible for radiation anomalies and the limitation of melt energy by the limited amount of sea ice. Prominent positive peaks in {MET} are found for the summers of 2011, 2012, and also 2010 but not so much for 2007, when ice melt seems to have stretched over a longer period compared to later years. All prominent extreme fall sea ice minima are followed by negative winter {MET} anomalies. This is due to the delayed onset of freezing in those years yielding anomalously strong winter ice production. More recent summers did not exhibit exceptional ice melt anomalies, with the summer of 2014 showing negative {MET} anomalies comparable to the early 2000s.

## 6 Seasonal Trends in the Arctic Energy Budget

**Figure 2:**
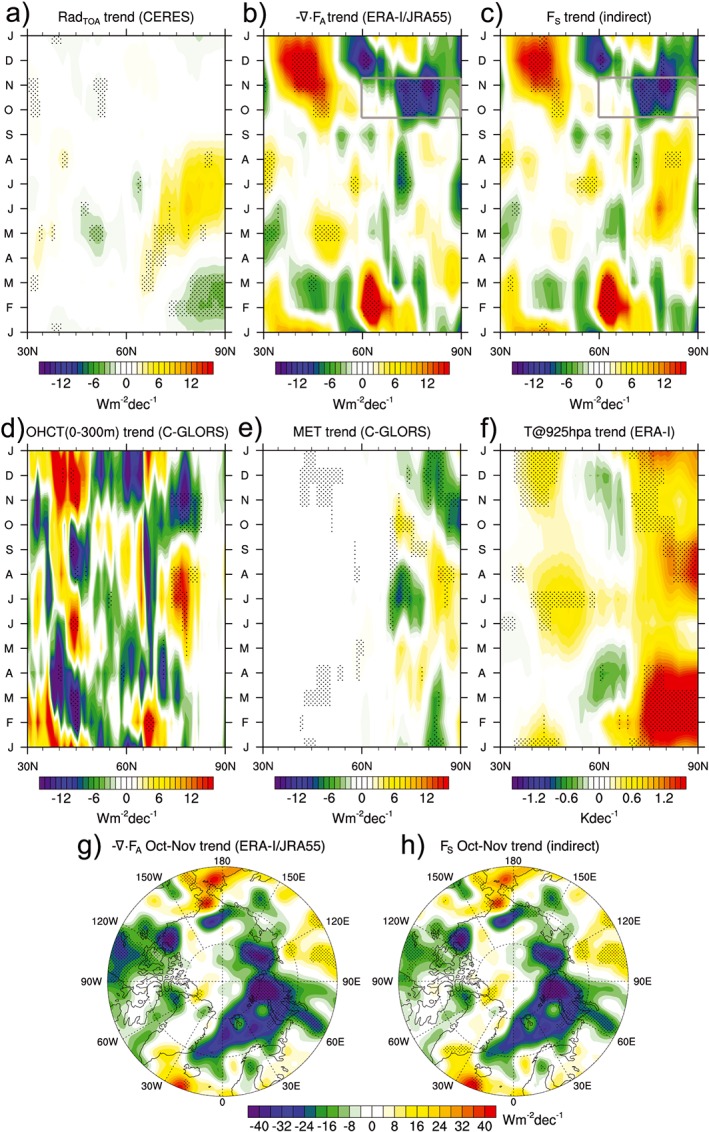
Zonally averaged seasonal least squares trends (March 2000 to February 2015) of (a) net radiation at TOA, (b) atmospheric energy convergence, (c) net surface energy flux (F_S_), (d) ocean heat content tendency (upper 300 m), (e) melt enthalpy tendency, and (f) air temperature at 925 hPa; maps of October–November trends of (g) atmospheric energy convergence and (h) F_S_ for the regions indicated in Figures 2b and 2c; stippling indicates significant trends at 95% confidence level.

In addition to the quite steady increase of Arctic Ocean energy content between 2000 and 2015, as discussed in section 3, recent changes of energy flows through and energy storage in the Arctic have a pronounced seasonal structure, as already noted in section 5. To investigate this further, we quantify these changes as zonally averaged seasonal linear trends (March 2000 to February 2015) of relevant fields over ocean (land masked out) in Figure 2.

As already documented by *Hartmann and Ceppi* [2014], trends in Rad_TOA_ from CERES (Figure 2a) show a pronounced seasonal cycle, with generally positive values in summer and a maximum around 80°N in June, i.e., near the climatological sea ice edge at the time of maximum insolation.

The structure of seasonal trends of − ∇·F_A_ (Figure 2b) is different. Trends are generally weak in summer, but a strong north‐south dipole of − ∇·F_A_ trends is present in October–November. Energy convergence north of 70°N decreased, while it increased south of 70°N. Temperature trends at 925 hPa (Figure 2f) are strongly positive north of 70°N year round as a result of the amplified surface warming in the Arctic [see, e.g., *Screen and Simmonds*, 2010]. This reduction in baroclinicity between 60°N and 70°N is consistent with trends toward reduced energy convergence in the Arctic.

Seasonal trends of F_S_ (Figure 2c) exhibit features both from Rad_TOA_ as well as − ∇·F_A_ trends. Positive trends are present at high latitudes in summer, consistent with positive Rad_TOA_ trends. During fall, F_S_ trends show a dipole pattern similar to − ∇·F_A_, i.e., trends toward increasing heat release from the ocean at high latitudes (north of about 70°N) and decreasing heat release from the ocean south of about 70°N. Summer trends are consistent with trends toward more open waters and consequently reduced albedo, while fall trends of F_S_ are balanced by reduced atmospheric energy convergence north of 70°N.

The observed trends could be related to either anthropogenic warming or some decadal oscillation, most notably the Arctic Oscillation (AO). The AO index indeed shows a positive yet insignificant trend, i.e., a trend toward lower Arctic sea level pressure, during fall over the 2000–2015 period (see Figure S5), consistent with the results of *Jaiser et al*. [2012]. However, a higher AO index is associated with increased storm activity in the Arctic, which is contradictory to the observed weakening of energy convergence (Figure 2b). This indicates that sea level pressure, i.e., the AO index, is not necessarily a reliable proxy for energy convergence in the Arctic, as the direction of the atmospheric flow and temperature gradients play a major role. A map of − ∇·F_A_ trends for October–November (Figure 2g) reveals that negative − ∇·F_A_ trends are concentrated along the ice edge, where the effect of sea ice retreat on surface energy flux (see Figure 2h) and consequently horizontal temperature gradients is largest. This is a clear sign that there is a strong thermodynamic component to the observed − ∇·F_A_ trends, which is not reflected in the AO index.

Seasonal trends of OHCT (Figure 2d) close to the sea ice margin (around 70°N to 80°N) are consistent with F_S_ trends, i.e., trends toward more positive OHCT (stronger ocean warming) during summer and more negative OHCT (stronger ocean cooling) during fall. Trends north of 80°N are close to zero as this region is largely ice‐covered year round which limits variations in vertical energy fluxes.

Energy going into ice melt exhibits seasonal trends which are complementary to OHCT trends north of 80 N (compare Figures 2d and 2e), i.e., trends are toward stronger ice melt in summer and stronger refreezing in fall/early winter. Visual inspection of trend maps (not shown) reveals that the phase shift of MET trends at lower latitudes (70°N–80°N) is related to trends toward earlier melt onset along the ice edges (April–May) which consequently reduces the sea ice available for melt in summer (June–August). The positive MET trends in fall, when climatological MET is negative, can be explained by weaker or a delayed onset of refreezing, consistent with trends toward a warmer sea surface and more heat release from the ocean in fall (Figure 2c) [*Markus et al*., 2009].

**Figure 3:**
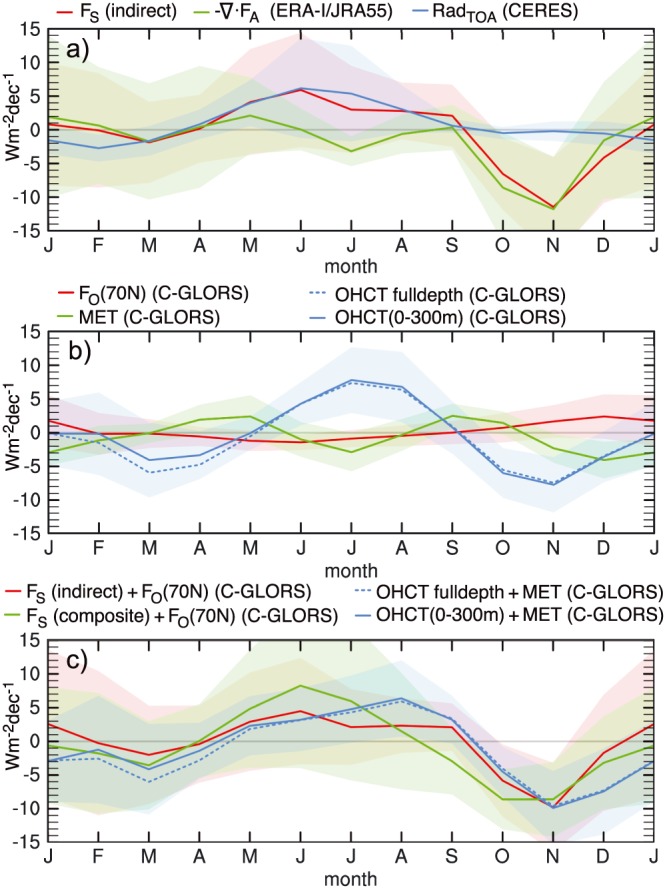
Arctic average least squares trends (March 2000 to February 2015) of (a) atmospheric energy convergence, net radiation at TOA, and net surface energy flux (F_S_), (b) melt enthalpy tendency (MET), ocean heat content tendency (OHCT), and ocean heat transport across 70°N (F_O_(70 N)), and (c) direct and indirect estimates of ocean energy tendencies based on OHCT + MET as well as the sum of F_S_ and F_O_(70°N). Shading represents 90% confidence intervals of the trends.

Seasonal trends of the area‐averaged polar cap energy budget (see equation (3)) are presented in Figure 3. Positive net surface energy flux trends during summer are linked to trends in net radiation at TOA, while negative F_S_ trends during fall are balanced by atmospheric energy transports (see Figure 3a).

Trends of {OHCT} are positive in summer, slightly lagging {F_S_} trends and negative during spring and fall (Figure 3b). Negative fall trends of {OHCT} are consistent with increased heat release from larger ice‐free ocean areas, while the negative spring trends are largely located in the North Atlantic and likely not directly related to changes in the Arctic (maps not shown). Seasonal trends of F_O_(70°N) are small. The results presented in Figures 3a and 3b are in good agreement with the climate model‐based results of *Tietsche et al*. [2011].

Area‐averaged trends of {MET} are positive in spring (April–May) associated with earlier melt onset (Figure 3b). Trends toward increased refreezing in the central Arctic and reduced refreezing at lower latitudes in fall (see Figure 2e) yield a positive area‐averaged MET trend. Winter {MET} trends are negative, suggesting a trend toward increased refreezing at that time of the year.

Three different estimates of seasonal trends in ocean energy storage rate are presented in Figure 3c. The estimate from the sum of {OHCT} and {MET} shows an amplification of the annual cycle whereby {OHCT} contributes the most (compare Figure 3b). Seasonal trends are similar when obtained indirectly from a combination of our two different {F_S_} estimates and F_O_(70°N). The differences between the curves in Figure 3c represent an estimate of the budget residual trend (compare equation (3)). Similar to *Balmaseda et al*. [2015], we compute the signal‐to‐noise ratio (SNR) of the results in Figure 3c as the fraction of the ensemble standard deviation and the ensemble mean, yielding an SNR of 2.6. We note that negligence of sea ice transports has only marginal impact on this result (compare results to discussion in section 2). Area‐averaged trend uncertainties shown in Figure 3 are generally large due to spatially compensating signals (see Figure 2), and the small sample size but several key results like the amplification of the annual cycle of {OHCT} are still significant and the SNR is reasonable. We also note that trends obtained from a median‐of‐pairwise‐slopes method are very similar to those discussed here (compare Figure 2 to Figure S6).

## 7 Summary and Conclusions

Different and largely independent observation‐ and reanalysis‐based data sets indicate that Arctic Ocean energy content increased at varying rates, whereby the average rate was about 1.1 (0.9, 1.2) Wm^−2^ for the 2000–2015 period. This value is very similar to the global mean OHC increases shown by the employed ocean data sets and also with the recent estimate of Earth's energy imbalance by *Llovel et al*. [2014]. Thus, the rapid warming of the Arctic (Arctic amplification) is confined to the surface and does not significantly increase the Arctic's energy imbalance above global average values.

Ocean heat transport seems to have contributed the most to the Arctic's energy accumulation prior to 2007, while positive radiative flux anomalies associated with the ice‐albedo feedback played an important role in more recent years. This suggests that anomalously strong oceanic energy import likely preconditioned Arctic sea ice for being susceptible for strong summer melt from 2007 onward.

The seasonal cycle of the Arctic energy budget has clearly amplified over the past 15 years. This is reflected in pronounced seasonal trends of {OHCT} and {MET}, which are largely balanced by trends in surface energy fluxes. Energy input into the Arctic Ocean during summer increased due to the ice‐albedo feedback. Energy release from the ocean in fall increased in return, largely balanced by reduced atmospheric energy convergence associated with weakened baroclinicity.

Although seasonal trends in Arctic Oscillation likely also played a role in modifying atmospheric energy transports, we found a clear thermodynamic component in the fall trends of atmospheric energy convergence. However, longer time series are required to more reliably separate trends from variability. Nevertheless, our results show that the Arctic climate system is able to efficiently remove the increased summer heat input during fall, leaving an average Arctic energy imbalance close to global mean values.

These results are largely in accordance with recent modeling studies [*Bintanja and Van der Linden*, 2013; *Carton et al*., 2015; *Tietsche et al*., 2011] but have not yet been documented based on observation‐constrained data sets. Moreover, our results imply that changes in poleward energy transport cannot be inferred from TOA radiation trends alone, as done, e.g., by *Hartmann and Ceppi* [2014]. This approach implicitly assumes constant energy storage rates in the Arctic, which is in disagreement with our results.

## Supporting information



Supporting Information S1
