# Supplementary material for: Facets of Arctic energy accumulation based on observations and reanalyses 2000–2015
Source: Geophys Res Lett. 2016 Oct 9;43(19):10420–9. doi: 10.1002/2016GL070557 (PMC5102146; doi:10.1002/2016GL070557)
Supplement: Supplementary file 1 — Supporting Information S1 [file GRL-43-10420-s001.pdf]

Geophysical Research Letters

Supporting Information for

Facets of Arctic energy accumulation based on observations and reanalyses 2000-2015

Michael Mayer[1, 2], Leopold Haimberger[1, 2], Marianne Pietschnig[1, 2], Andrea Storto[3]

[1] Department of Meteorology and Geophysics, University of Vienna, Vienna, Austria

[2] Austrian Polar Research Institute, Vienna, Austria

[3] Centro Euro-Mediterraneo sui Cambiamenti Climatici, Bologna, Italy

Contents of this file

Text S1
Figure S1
Figure S2
Text S3
Figure S3
Text S4
Figure S4
Figure S5
Figure S6

Introduction

A rationale for the choice of radiation data for the composite of surface energy flux is given in S1. The strong annual cycle in the number of observations going into EN4 is presented in Fig. S2. Temporal homogeneity of sea ice fraction and thickness from PIOMAS, C-GLORS, and two satellite products are assessed in S3. Accumulated energy fluxes into the Arctic along with Arctic energy changes are discussed in S4. Seasonal trends of the Arctic Oscillation index for the periods 1979/03-2015/02 and 2000/03-2015/02 are shown in Figure S5. Figure S6 is analogous to Figure 4 in the main text, but here trends are computed with a median-of-pairwise-slopes method.

Text S1

Surface net longwave radiation from CERES exhibits a major discontinuity around end of 2007 (see Figure S1). This may be related to changes to temperature and humidity input to the radiative transfer model associated with the change from GEOS-4 to GEOS-5 in January 2008 (see data quality summary under http://ceres.larc.nasa.gov/documents/DQ_summaries/CERES_EBAF-Surface_Ed2.8_DQS.pdf for details). ERA-Interim longwave radiation seems more stable than the CERES fluxes.

Shortwave fluxes from CERES appear to be sufficiently stable (not shown) and hence are employed for our surface energy flux composite.

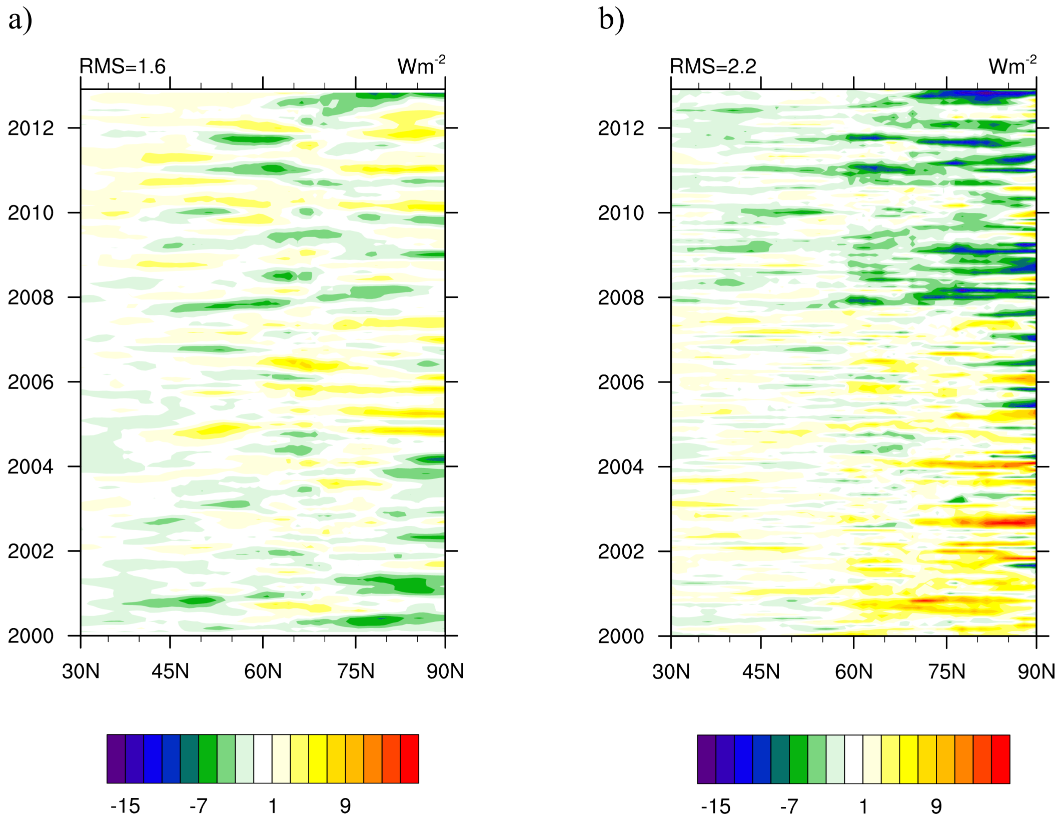

Figure S1 Monthly anomalies of net longwave radiation at the surface from a) ERA-I and b) CERES-EBAF 2.8.

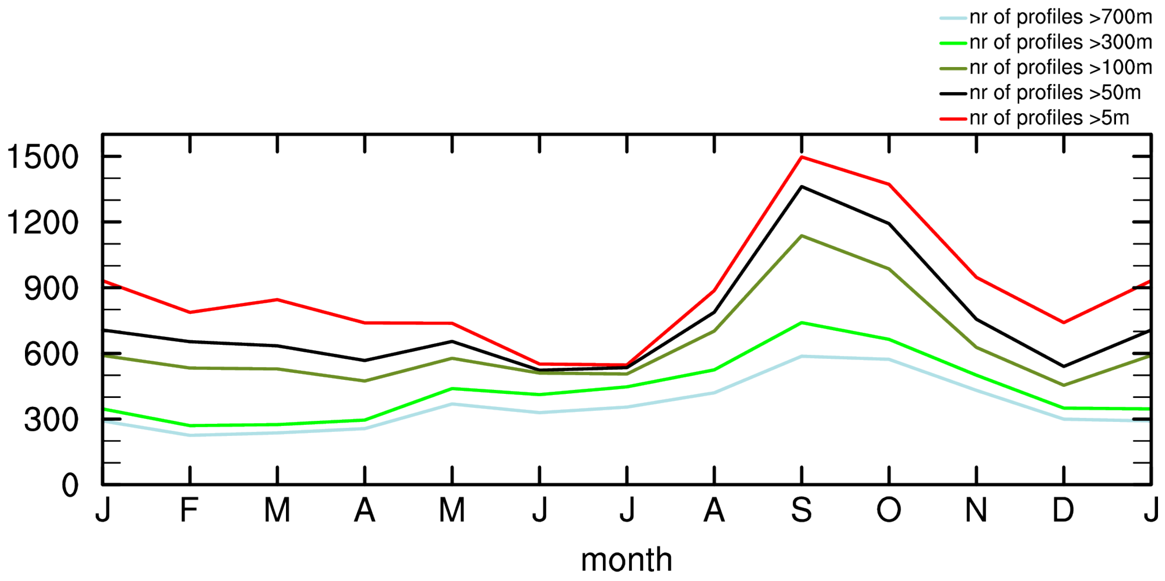

Figure S2. Mean annual cycle (2000-2014) of number of observations going into EN4 north of 70N as a function of maximum depth of the respective observations. Units are observations per month.

Text S3

Sea ice fraction data assimilated in PIOMAS and C-GLORS are not strictly independent, since they are derived from measurements of the same satellite instruments. However, PIOMAS assimilates the "Near-real-time Ice and Snow Extent" product (Nolin et al. 1998), while C-GLORS uses "Sea Ice Concentrations from Nimbus-7 SMMR and DMSP SSM/I-SSMIS Passive Microwave Data" (Cavalieri et al. 1996) (both provided by the National Snow and Ice Data Center). The latter is designed to provide consistent time series, while the former is not. We now compare sea ice concentrations from C-GLORS and PIOMAS to the "NOAA/NSIDC Climate Data Record of Passive Microwave Sea Ice Concentration" sea ice concentration data set, which is designed to provide homogeneous sea ice time series from 1978 onwards (Peng et al. 2013).

There is a clear shift in the NOAA/NSIDC minus PIOMAS differences of summer sea ice fraction anomalies around 2008/09 (Fig. S3a). This shift is not present in the NOAA/NSIDC minus C-GLORS differences of summer sea ice fraction anomalies (Fig. S3b). Similar results are obtained when comparing PIOMAS and C-GLORS to reprocessed Ocean and Sea Ice Satellite Application Facility (OSI-SAF; Andersen et al. 2007) satellite data (not shown). These results indicate that PIOMAS (summer) sea ice fraction indeed exhibits a temporal discontinuity around 2008/09.

Sea ice thickness in C-GLORS is weakly relaxed towards that of PIOMAS. However, assimilated sea ice fraction directly affects effective sea ice thickness in PIOMAS, but not sea ice thickness itself (Lindsay and Zhang 2006; effective sea ice thickness means the grid-box average sea ice thickness). Hence, it is not surprising that the shift in the C-GLORS minus PIOMAS differences of summer effective sea ice thickness (Fig. S3c) around 2008/09 is qualitatively similar to the shift in PIOMAS sea ice concentrations (Fig. S3a). We conclude that the shift in the (summer) thickness differences between C-GLORS and PIOMAS is due to the temporal inhomogeneity in PIOMAS sea ice fraction. Hence, we view effective sea ice thickness and consequently sea ice mass from C-GLORS as a more reliable data source for seasonal changes in sea ice melt over the considered period.

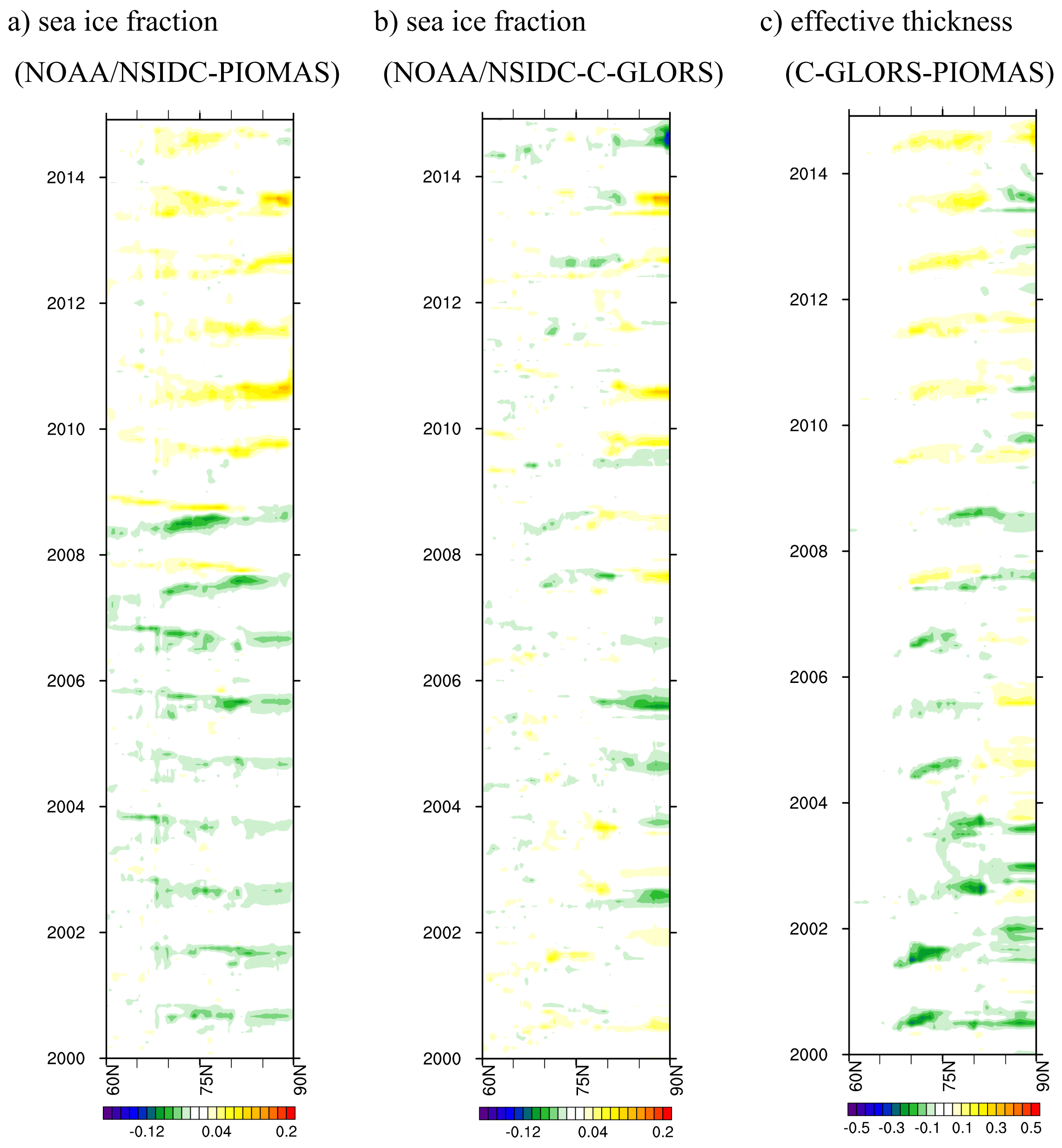

Figure S3 Difference in monthly anomalies of sea ice fraction between a) NOAA/NSIDC and PIOMAS data and b) NOAA/NSIDC and C-GLORS data. Differences in monthly anomalies of effective ice thickness between C-GLORS and PIOMAS are shown in c). Units in c) are meters.

Text S4

The sum of net surface energy flux and ocean heat transport across 70N should add up to give the energy storage in the Arctic ocean (long-term atmospheric storage rate is small; see eq. 3). However, although the sum of $\{Rad_{TOA}\}$, $\{-\nabla \cdot F_A\}$, and $\{-\nabla \cdot F_O\}$ agree remarkably well ($-116 Wm^{-2}$, $93 Wm^{-2}$, $17 Wm^{-2}$, respectively) with a small residual (after including $\{-\nabla \cdot F_I\}=0.5 Wm^{-2}$ based on C-GLORS), this indirect estimate does not match the observed storage rate. Hence, as a less strict check, we compute monthly anomalies of the flux convergence into the Arctic ocean ($=\{F_S\}-\{\nabla \cdot F_O\}$), add the 15-year average storage rate obtained from the increase of {OHC} and {ME}, and then accumulate these flux convergence anomalies. Energy increase in the Arctic based on various datasets (as in Fig. 1a in main text) along with accumulated energy fluxes into the Arctic are presented in Figure S4. Variations of total ocean energy agree remarkably well with the convergence estimate using JRA55 atmospheric transports and also the estimate using ERA-I (except for a constant offset between 2001 and 2011). For example, the strong increase of ocean energy during 2011-2012 is shown in a very similar manner by both accumulated convergence estimates.

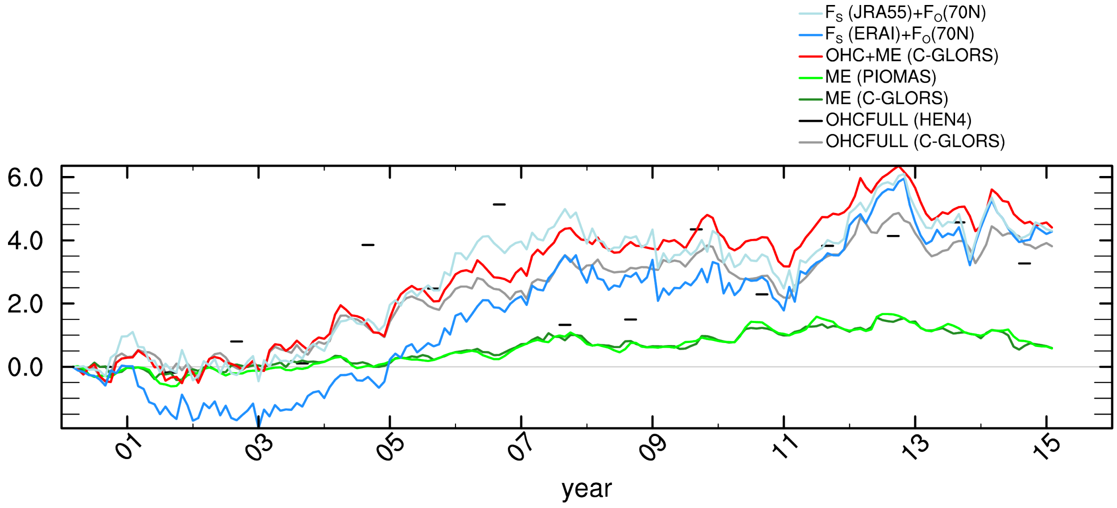

Figure S4 Energy increase in the Arctic (ocean-covered area north of 70N) as estimated from storage terms and accumulated energy estimated from F_S and F_O(70N) (units are 10^8 Jm^{-2});

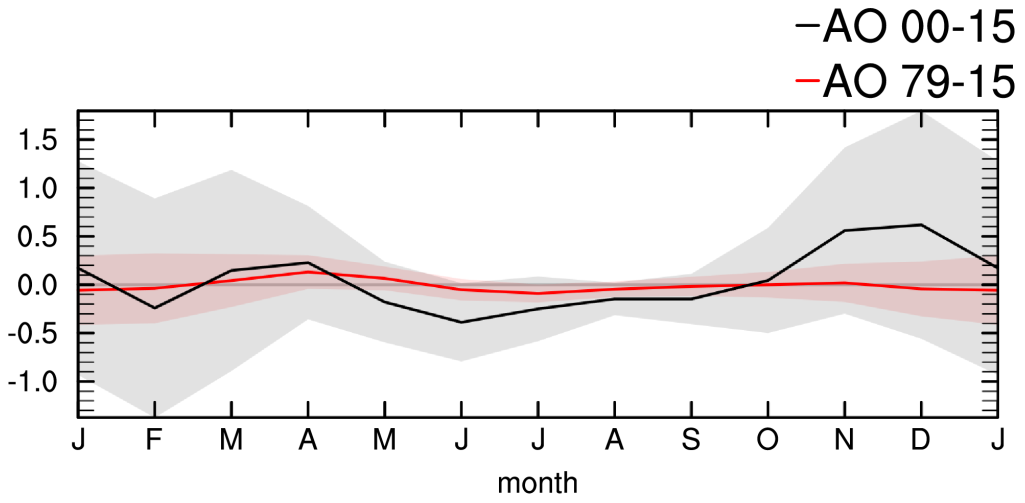

Figure S5 Seasonal linear trends of Arctic oscillation index over 1979/03-2015/02 (red) and 2000/03-2015/02 (black). Units are decade^{-1}. Shading represents 90% confidence intervals of the trends. Data obtained from the National Oceanic and Atmospheric Administration under http://www.cpc.ncep.noaa.gov/products/precip/CWlink/daily_ao_index/monthly.ao.index.b50.current.ascii.table

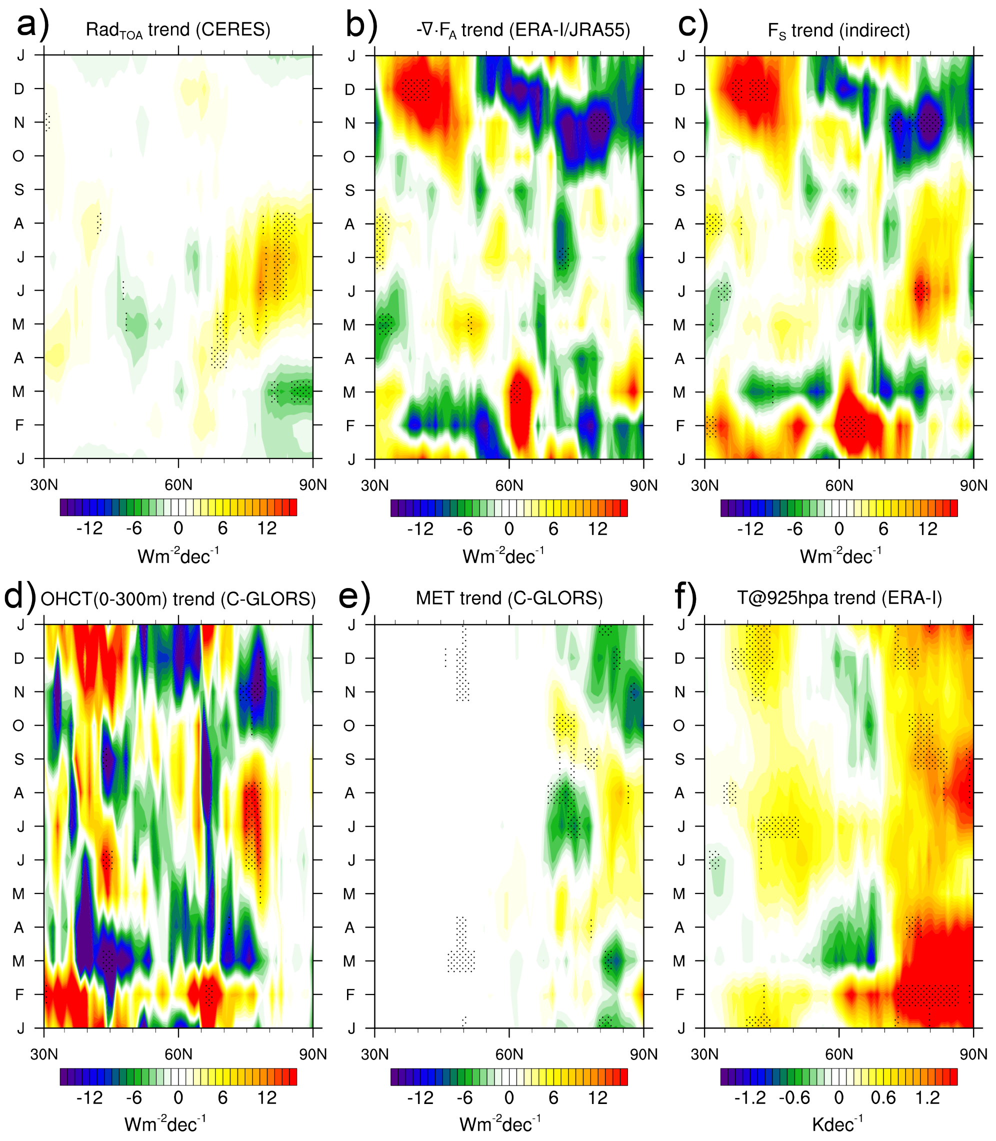

Figure S6 Zonally averaged seasonal median-of-pairwise-slopes trends (2000/03-2015/02) of a) net radiation at TOA, b) atmospheric energy convergence, c) net surface energy flux (F_S), d) ocean heat content tendency (upper 300m), e) melt enthalpy tendency, and f) air temperature at 925hPa; stippling indicates significant trends at 95% confidence level.
